# Pedagogical Function of Referees in Youth Sport: Assessment of the Quality of Referee–Player Interactions in Youth Soccer

**DOI:** 10.3390/ijerph17030905

**Published:** 2020-02-01

**Authors:** Wiesław Firek, Katarzyna Płoszaj, Marcin Czechowski

**Affiliations:** Faculty of Physical Education, Józef Piłsudski University of Physical Education in Warsaw, 00-968 Warsaw, Poland; katarzyna.ploszaj@awf.edu.pl (K.P.); marcin.czechowski@awf.edu.pl (M.C.)

**Keywords:** referee, referee–player interactions, soccer, assessment, observational instruments, educational practice, pedagogical function

## Abstract

We assume that all institutions and individuals involved in the organization of sport for children and young people should utilize the educational potential of sport. We assessed the quality of referee interactions with children during sports competitions in soccer. Based on the developmental theory and research suggesting that interactions between kids and adults are the primary mechanism of their development and learning, we focused on the quality of the referee–player interactions in terms of (1) emotional support, (2) game organization, and (3) instructional support. Twenty-five soccer referees who refereed matches for children aged 9–12 years were recruited. The Referee Educational Function Assessment Scoring System (REFASS) was used to assess the quality of the referee–player interactions. This tool was developed based on Classroom Assessment Scoring System—Upper Elementary. Regarding the REFASS dimensions, the mean scores for positive climate, Sensitivity, behavior management, content understanding and quality of feedback were in the medium range, while productivity and negative climate in the high range. In the case of the positive climate variable, the lowest mean ratings were recorded compared to other assessed dimensions. The assessments of the quality of referee–player interactions obtained for particular dimensions translated into the ratings for the specified domains. The highest ratings were given to game organization (6.0 ± 0.8; Me = 6.0), whereas the emotional support and instructional support were in the medium range (4.6 ± 1.5; Me = 4.5, and 5.2 ± 1.8; Me = 6.0, respectively). Referees are usually not aware of their pedagogical function and the complexity of their respective responsibilities. They are commonly considered to be ordinary technicians and evaluators of performance in competition. Based on the results, a postulate was formulated that referees should consciously perform a pedagogical function in the youth sport. Therefore, it is necessary to train them in educational methods and techniques appropriate to the age and needs of the child. The referees will then be prepared to take actions to prevent negative behavior of players on the field and to encourage prosocial behavior.

## 1. Introduction

The lack of empirical data to support theories that emphasize the educational role of sport and the results of studies pointing to its anti-educational effect make the problem of the links between education and sport a subject of constant scientific and social debate. The conclusions formulated on this subject point to three types of relationships: (1) sport educates, (2) sport is neutral in its educational aspect, and (3) sport is anti-educational [1]. The vast majority of researchers believe that sport has great educational potential. It is often stressed that participation in sport increases children’s self-esteem [2], helps maintain emotional balance, and encourages cooperation and leadership [3,4]. Training, sports competition, and losing and winning can be a rich source of positive personal and social experiences provided that these situations are skillfully and consciously used for educational purposes [5].

Those who think differently about the educational role of sport believe that if sport has any effect on education, it is negative. Such conclusions were confirmed not only by empirical research [6,7] but also by daily observation of the world of sport, with many examples of fraud, doping, bribery and objectification of athletes.

The discussion on the relationship between sport and education is complemented by those who believe that sport neither helps nor hinders the achievement of educational goals, as these are separate and independent areas. The experience gained in sport is so different from that gained in everyday life that it does not provide any basis for the development of traits that can be used outside sport [6,8].

Let us assume, however, that sport, which is an important area of activity for children and young people, can and should be used to instill social attitudes and behavior. It is worth noting here that the involvement of children in sport does not automatically produce educational benefits for them [9,10]. The potential benefits depend on the actors who should consciously influence the quality of the interactions taking place in the sport environment. Only then can the sports environment become an intentional educational environment.

In educational relations, there is a distinction between the educating and educated entities. Sport first educates competitors taking part in a competition, and then the audience gathered at the stadium and those who watch sporting events through the media [7,8,11]. The identification of entities being educated is not a problem. But who at the sports stadium plays the role of the educator? The pedagogical literature on children’s and youth sport has already made multifaceted analyses of the role of coaches [5,12,13,14,15] and parents [16,17,18,19,20]. In their research, sports educators take into account not only the perspective of the coach and the parent but also the perspective of the child. This is in line with current research trends in physical education and sport in order to focus not only on teaching (teacher, trainer and parent activities) but also on learning (pupil, competitor). Quennerstedt, Öhman, & Armour [21] stressed that learning is the essence of pedagogy and sport coaches play an important role as teachers. It should be remembered, however, that there is also a referee on the sports field, who is rarely called an educator [22]. The question can be asked: Why is the pedagogical function of a referee often overlooked in youth sport? A sports referee has been the subject of numerous and multidimensional studies, but few of them have focused on the educational role of referees.

Most publications on the referees have mainly focused on: (a) the determinants of their decisions [23,24,25], (b) the psychological burden on referees and the number of decisions they make [26,27], (c) stress and insults they are exposed to [28], (d) expected physical fitness in referees [29], and (e) identification of the key competencies of the referee [30,31]. A referee making about 200 decisions in a soccer match [26] can and should take into account the educational objective of his or her actions.

There are theoretical justifications for enriching the referee’s work with an educational dimension [32], but there is still a lack of empirical studies on the interaction between referees and players and the importance of these interactions for the multilateral development of young athletes. This study represents an attempt to supplement the existing knowledge on the role of the referee on the sports field. Therefore, the main research problem in this study is the question of the pedagogical function of the referee in the youth sport and the quality of his or her interaction with children during soccer matches. The results of the research may be of interest to sports associations that organize sports competitions for children and youth, train referees and directly supervise the quality of their work.

## 2. The Pedagogical Function of the Referee

The modern concept of positive youth development through sport [10,33] proposes to redefine sport objectives so that they are not result-oriented but stimulate a positive experience based on enjoyment of participation in sport and play [34,35,36]. If it is not possible to take such objectives into account, the focus should be at least on balancing achievement, participation and personal development for the youngest (Developmental Model of Sport Participation - DMSP), especially up to the age of 13 [37]. While in professional sport the educational function is limited, it is extremely important in children’s and youth sport. The benefits of referees’ pedagogical work for the multilateral development of young players and instilling appropriate social attitudes of spectators, coaches, and other sports activists are highlighted [16,22]. Hence, a referee should be seen not only as a sports competition enforcer [38,39,40] but also as a teacher with adequate knowledge of pedagogy and developmental psychology [41]. Most research findings on the referees’ use of such techniques as modelling, adopting child perspective, redirecting inappropriate behavior, building a positive climate, etc. proved that using those techniques increase the effectiveness of educational effects [34,35,36]. The literature also draws attention to the desired competencies and characteristics of a referee and stresses that this should be a person whose authority on the field results not only from the function performed (authority vested in them) but also from his or her knowledge, skills and attitudes that inspire social recognition [30].

Assuming that sport is a social practice and represents a social system in itself, of which the educational system is an integral part [32], we consider that the functions of a referee must also be analyzed from the pedagogical perspective. Consequently, refereeing in youth sport should be regarded as a social activity having essentially a pedagogical dimension, whose importance is not sufficiently recognized and understood by society at present due to the traditional model of a referee. In addition, based on the results of research and theoretical considerations about the role of referee in youth sport, we recognize that the pedagogical function of a referee in youth sport can be performed on two levels. The first level results from the rules of the game, which are not devoid of educational values. The rules of the game usually prohibit unsportsmanlike conduct and require respect for the opponent. Enforcing these principles is an educational activity, as is teaching young players the rules of the game or their interpretation. This level can be called minimalistic (formal dimension of the educational function of a referee). At the second (higher) level, the referee, who is aware that he or she represents an important link in sports education, should start to apply a broader range of educational measures, not recorded directly in the rules of the game (informal dimension of the educational function of a referee). The referees in youth sport should use their position to build a positive climate to ensure that young players feel safe and the field seems to be a friendly environment to them [10,33]. Referees, depending on the level of psychophysical development of the player, should use the following verbal and non-verbal messages: smiles; positive comments such as ‘Bravo!’ ‘Well done! etc.; an enthusiastic attitude; a kind tone of voice; polite phrases; and eye contact [42]. Furthermore, the referee should respond to the needs of the player, answer the player’s questions (explaining decisions), recognize and accept the emotions of the players (e.g., crying, sadness), resolve conflicts constructively, establish physical contact, and instruct (by explaining, describing, or presenting the correct way to perform the task, etc.). Scientists postulate that positive climate approaches should be introduced into children’s sports during a variety of tournaments and competitions [43,44,45]. Morris and O’Connor [30] stressed that referees are aware that a key skill in their work is to establish positive relationships with players. The referee should avoid attitudes characterized by irritability, nervousness, anger, harshness, sarcasm, shouting, threatening, hurtful remarks, humiliation, offensive and disrespectful gestures, expressing criticism in an unconstructive manner etc. In addition to the above-mentioned positive climate and sensitivity to the needs of the players, termed emotional support [42], the referee should show skills of game organization. Game organization does not mean influencing the course of the competition or the final score. This is more about managing behavior. The referee should be proactive and prevent negative behavior and, if it occurs, effectively redirect it, solve problems quickly and prevent their escalation and continuation. In this context, the referees are the first and last ‘line of defense’ in maintaining the playing environment that is conducive to education.

A referee should skillfully exploit the educational potential of sport and consciously perform an educational function at both formal and informal levels mentioned above. In youth sport, it is the referee who influences the psychosocial experiences of players, making his or her decisions and behavior an important source of educational stimuli.

## 3. Purpose

Assuming that all actors involved in the organization of sport for youth should use its educational potential, the aim of the work was to assess the quality of referee interactions with children aged 9–12 years in soccer competitions.

An interesting experiment was presented in a study by Arthur-Banning, Paisley, & Wells [41] who examined the effect of the use of prosocial behavior techniques by referees on the attitudes of young basketball players. The study revealed that in matches officiated by referees trained in the use of these techniques, significantly more instances of sportsmanlike behavior were observed among players compared to matches refereed by referees not trained in these techniques. In our study, we attempted to present a wider range of the referee’s pedagogical influence on players, including not only building a positive climate and strengthening prosocial behavior, but also the ability to redirect negative behavior, the referee’s sensitivity to the emotional, social and cognitive needs of the players, and instructional support.

## 4. Materials and Methods

The research was conducted in the Mazowieckie Voivodeship (Poland). Based on the developmental theory and research suggesting that interactions between kids and adults are the primary mechanism of their development and learning, the study focuses on the referee–player interactions in terms of (1) emotional support, (2) game organization, and (3) instructional support. The research is part of a broad discussion on the determinants and methods of increasing the effectiveness of physical education and youth sport.

The study covered 25 soccer referees (24 male and 1 female) who have refereed matches for children aged 9–12 years. The referees were licensed by the Mazovian Soccer Association for the 2018/2019 season and refereed boys’ matches (Orliki and Młodziki categories). The referees were aged 29.64 years (SD = 14.26) with refereeing experience ranging from 1 to 38 years (M = 6.84, SD = 10.43).

The referees were observed as they officiated, during the period of the study, soccer matches of children in the Mazowieckie Voivodeship in Poland. The games were organized by district sports associations. When selecting referees, their sex, age, work experience, and refereeing certification were not taken into account since performing an educational function in sport requires specific knowledge, skills and attitudes regardless of the above-mentioned characteristics. Due to the need to record verbal and non-verbal communication of the referee, the observations of matches of players aged 9 to 12 years were undertaken. Younger children often played matches without a referee, while older soccer players played on large fields, which significantly limited effective observation.

Previous empirical studies concerning referees have usually used a diagnostic survey method, asking referees for their opinions, self-assessment or expert assessment. By asking a respondent questions, the interviewer probably influences the answers and perhaps strengthens the respondent’s position by forcing him or her to declare opinions [46]. It follows that the answers to the referees’ questionnaire items should be approached as declarations. They may differ from the actual actions on the field, where emotions, contact with players and pressure associated with the need to make quick decisions appear. In order to eliminate the subjective responses of the referees, it was decided that a live observation method would be used to measure the quality of the referee–player interactions. However, in sports sciences, there is no ready-made tool enabling reliable observation of the pedagogical work of a referee. The sports associations also failed to develop a system of evaluation and control of the referees’ work in the pedagogical dimension. Furthermore, research on the educational function of a referee in the sport for children and adolescents is exploratory in nature, attempting to shed light on this issue, and requires the development of appropriate research tools. Therefore, tools to assess the quality of the referees’ interactions with the players were sought in the field of pedagogy.

The Classroom Assessment Scoring System—CLASS [42] is among the most current and widely used standardized assessment of social and instructional interactions in classrooms. Based on CLASS —Upper Elementary system, the Referee Educational Function Assessment Scoring System (REFASS) was developed. The REFASS tool includes all the domains contained in CLASS, i.e., Emotional Support, Classroom Organization (renamed Game Organization), and Instructional Support. The dimensions and indices of referee–player interactions have been reduced and context-specific to the referee’s work and the sports environment. The focus was on the key tasks of the referee for the emotional, social and cognitive development of children aged 9–12 years.

**Figure 1:**
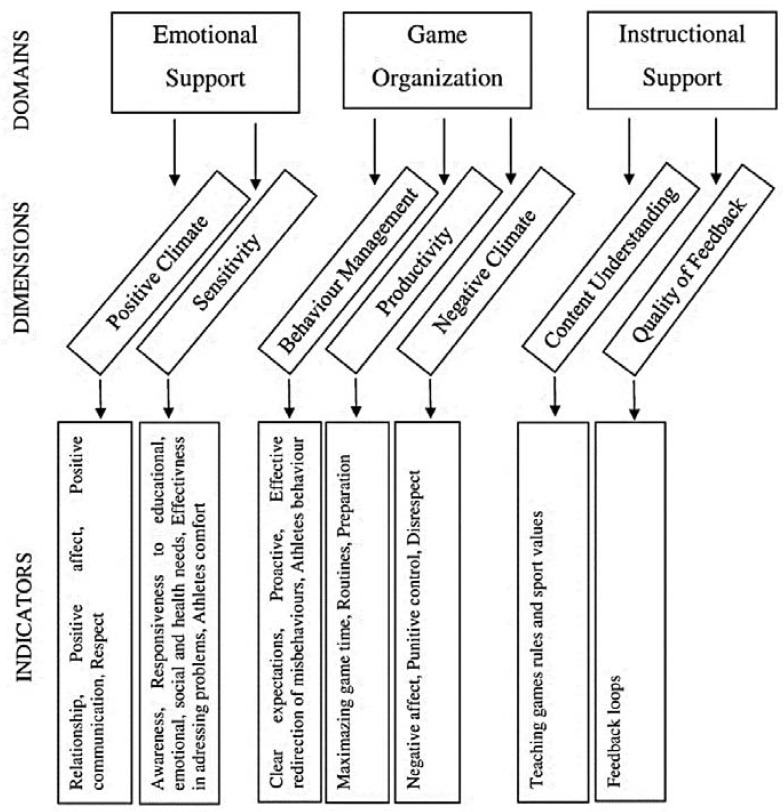
Framework for assessing quality referee–player interactions.

At the broadest level, interactions between referees and players have been grouped in the examinations using the REFASS tool into three domains (Figure 1).

1. Emotional support. This domain consists of two dimensions. The first dimension is *positive climate*, reflecting the emotional connection and relationships between referee and athletes, and the warmth, respect, and enjoyment communicated by verbal and non-verbal interactions (Table 1). The second dimension is *referee sensitivity,* which reflects the referee’s timely responsiveness to the educational, social, emotional, health, behavioral developmental needs of individual athletes and the entire team.
2. Game Organization. This domain contains three dimensions: *behavior management*, *productivity* and *negative climate*. Behavior management encompasses the referee’s use of effective methods to encourage desirable behavior and prevent and redirect misbehavior. Productivity takes into account time management and procedures in order to optimize game time (e.g., minimization of breaks in the game). Negative climate reflects the overall level of negativity among referees and athletes in the sports fields; the frequency, quality, and intensity of referees and athletes’ negativity are important to observe.
3. Instructional Support. This domain consists of two dimensions: *content understanding* and *quality of feedback.* Content understanding refers to the players’ understanding of the referee’s messages related to the rules of the game and the values of sport. Quality of feedback indicates how the referee communicates his or her game decisions, extends players’ knowledge and dispels their doubts about the rules of the game and the referee’s decisions. The focus of this dimension is on the gestures and signals and verbal messages of the referee.

Observers carefully reviewed the description and made judgments on the following instruction (Table 2).

The domain scores are simply the average dimension scores across each corresponding domain (emotional support: positive climate and sensitivity; game organization: behavior management, productivity and negative climate (reversed); instructional support: content understanding, quality of feedback)

The advantage of this tool is that it can be used in all sports, especially team sports, where the interactions between referee and players are more frequent and the referee has a greater effect on the course of the game than in e.g., track and field events or rhythmic gymnastics.

## 5. Procedures

The main examinations were preceded by a consultation of the tool with representatives of the Referee Department of the Mazovian Soccer Association. The result of this consultation was the adaptation of the tool to the specifics of the referee’s work.

The sports association provided the referees and organizers with information on the research. The observation took place in a controlled manner, i.e., it consisted in regular live observation of the referee–player interactions according to a strictly defined REFASS key. This allowed for minimization of the subjective assessment of the observer and the statistical analysis of the research results.

The data was collected by trained supervisors (authors of the paper). The main examinations were preceded by pilot research consisting in observation of the interactions between referees and players during three matches. The pilot research also served to clarify and standardize the criteria for observer assessments. It was assumed that the observers’ agreement on the results of the observation should be at least 75%. In the main research, each match was observed by one observer who noted the referee–player interactions throughout the match by completing a score sheet. They also took notes that were helpful in establishing the final assessment of each dimension and domain. After the match, according to the recommendations of the creators of CLASS, the observer derived numerical ratings for all of the REFASS dimensions. These ratings were based upon the observer’s knowledge of the dimension definitions, markers, and the written notes the observer has made during the entire observation window for each dimension [42].

## 6. Analytic Strategy

Basic statistical measures were used to describe the results: arithmetic means, standard deviations, and medians. The significance of differences was assessed for the three domains. Due to severe departures from normality (ascertained by the Shapiro–Wilk test), Friedman’s ANOVA and then Wilcoxon’s signed ranks test were used to determine significance. The Cronbach’s alpha coefficient was calculated to assess the consistency of analyzed variables. Exploratory factor analysis (EFA), with principal axis factoring (PAF), was used to identify the underlying relationships between variables. The Kaiser–Meyer–Olkin measure of sampling adequacy (KMO) and the Bartlett’s test of sphericity were run to check that the data were appropriate for EFA. An orthogonal Varimax rotation was used for factor rotation. For item reduction, the cut-off for significance of factor loading was set to 0.6. Calculations were performed using the STATISTICA v. 10.0 statistical software package (StatSoft, Tulsa, OK, USA). In all analyses, the level of significance was set at 0.05.

## 7. Results

Means and standard deviations for all examined dimensions are reported in Table 3. Regarding the REFASS dimensions, the mean scores for positive climate, sensitivity, behavior management, content understanding and quality of feedback was in the medium range, while productivity and negative climate in the high range. In the case of the positive climate variable, the lowest mean ratings were recorded compared to other assessed dimensions.

**Figure 2:**
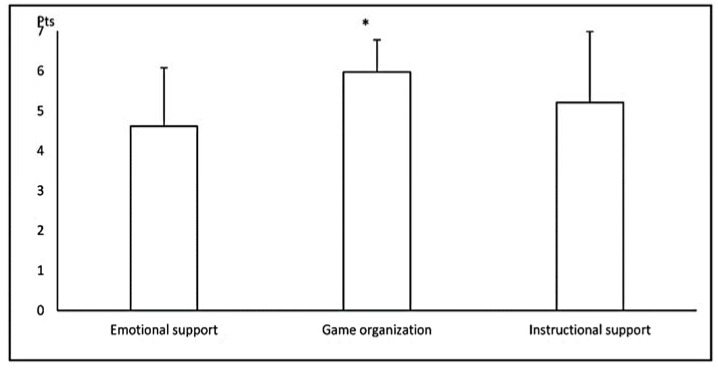
Mean (±SD) ratings of the quality of referee–player interactions in individual domains observed in Soccer (n = 25) referees. * significantly (*p* < 0.05) higher than in the two other domains (Friedman’s ANOVA followed by Wilcoxon’s signed ranks test).

The assessments of the quality of referee–player interactions obtained for particular dimensions translated into the ratings for the specified domains (Figure 2). The highest ratings were given to game organization (6.0 ± 0.8; Me = 6.0), whereas emotional support and instructional support were rated significantly lower and were both in the medium range (4.6 ± 1.5; Me = 4.5; *p* < 0.001, and 5.2 ± 1.8; Me = 6.0; *p* = 0.025, respectively).

The value of Cronbach’s alpha of 0.827 obtained from the analyses indicates high similarity of the assessments taken into account in the study. The lowest values of correlation with the overall result were found for the negative climate and productivity variables (0.278 and 0.367, respectively). These items lowered the consistency of the entire scale whereas their removal led to an increase in Cronbach’s alpha to 0.831 for negative climate and to 0.832 for productivity. Other ratings correlated much more strongly with the total scale, with the coefficients ranging from 0.570 (behavior management) to 0.776 (content understanding).

Factor analysis was made for seven variables. The KMO measure was at an acceptable level (0.695) and Bartlett’s sphericity test was also significant (*p* < 0.05), indicating the validity of the factor analysis. In the initial phase of factor analysis, two eigenvalues were ≥1, thus meeting the Kaiser criterion when determining the number of factors. However, no clear and easy in interpretation structure was obtained for the two-factor solution, explaining only 66.4% of the variance (Table 4). Therefore, considering small number of variables included in the analysis, in the second approach the number of factors was determined using Cattell’s criterion (scree plot) and the percentage of explained variance (> 0.75). In this solution, three factors were identified, described in details below.

The results of the exploratory factor analysis (EFA) including three factors partially confirm the belonging of individual assessments to the separated domains. After the rotation, Factor 1 and 2 corresponded to the emotional support and instructional support domains, respectively. However, three assessments that constitute the game organization domain were spread across three factors; the third factor contained only a variable negative climate with high factor loading (>0.9; Table 5). This indicates, similarly to the results of the analysis of Cronbach’s alpha, a slightly different character of this variable compared to other assessments. For all seven variables the simple structure criterion was met, the majority of them represented high communality estimates (>0.79); slightly lower values were observed for sensitivity and behavior management (0.622 and 0.690, respectively). The three-factor solution explained about 78.6% of the total variance.

## 8. Discussion

There is a strong need in educational practice in sport to measure educational influences of referees [22,32]. Based on the assumption that the referees should consciously participate in educational practice in competitive youth games, and assuming that the referee–player interactions are the basic mechanism of the referee’s educational influence, the aim of the research was to assess the quality of referees’ interactions with children in soccer in terms of emotional support, game organization and instructional support.

The findings from this study suggest that the three-factor structure partially confirm the belonging of individual assessments to the separated domains. Emotional support and instructional support were well identified. Negative climate has a different character compared to other assessments, but according to Hafen et al. [47] it should be integrated in the organization domain because as when negative climate is at a high level, there tends to be disruption to both the behavior management and the productivity. Hamre et al. [48] presented results on various approaches for factor analysis and factor structure in an effort to understand the organization of teacher–student interactions in secondary classrooms. They analyzed a one-factor structure that loads all dimensions on a single factor, a two-factor model that combines emotional support and instructional support into one factor while leaving classroom organization as a separate factor, and the original three-factor model. We have decided to use the three-factor model because it fits the natural variation in observed referee–player interactions in comparison with a single-factor or two-factor model. The REFASS was developed based on CLASS-UE [42], an extensive literature review on sport pedagogy and expert consultations (Referee Department of the Mazovian Soccer Association), who have agreed that REFASS measures important aspects of referees’ work, suggesting construct validity. This study confirms that referee–player interactions can be reasonably organized into three domains.

### 8.1. Emotional Support

In our research, the quality of interactions between referees and players in the domain emotional support was rated as medium (M = 4.6). The medium rating means that the referees and some players showed mutual interest, enjoyed interactions during the match, were in physical proximity, laughed and were excited in the same moments of the competition. However, the referees did not utilize the full potential of creating a friendly atmosphere. Trudel et al. [27] presented data showing that referees in professional sport spent 44.7% of their game on average on monitoring players without interactions, with an additional 13% waiting for playing. The referees in youth sport should be much more attentive to the quality of their interactions with athletes in order to build a positive climate which, especially for the youngest athletes, facilitates instilling appropriate behavioral norms [41,49].

Collins and Barcelona [50] postulate that administrators, parents and coaches should focus on developing athletes and creating a positive sports experience by increasing the pleasure of sports competition, developing positive support and cultivating relationships. The same conclusion came from Andersson’s research [22]. Referees should balance competitive seriousness and the spirit of play so that they can supporting players’ educative experiences and promotes enjoyment, inclusion and equality, respect and learning. It will allow a positive atmosphere to arise, where winning by any means necessary is replaced by doing one’s best, learning and developing. A referee who is present on the field during a match has much more possibilities to stimulate positive emotions and sporting experiences of players than coaches, supporters or administrators staying outside the playing area.

Positive verbal and non-verbal communication and the enthusiasm of the educator help release positive emotions of the child and thus lead to the faster and easier acquisition of new skills, knowledge and experiences [42,51]. During the study, it was observed that the referees’ attempts to verbally or non-verbally influence players in order to relieve tension or cause certain changes in their behavior were often ignored by players, especially when these messages were short and superficial or incomprehensible to the competitors due to their age, whereas non-verbal actions were illegible as to their intentions.

The quality of referees’ interactions with competitors in terms of the referee sensitivity dimension was assessed as medium. The referees generally monitored the situation on the field and were sensitive to the players’ needs for additional support in health-related situations (M = 5.2). Most often, they did not notice the signals related to the cognitive, emotional or social needs of the players. Even if the referees noticed and tried to solve the problems, they did not verify in the long term whether their actions brought about positive effects in the form of satisfying the specific needs of the child. The players sometimes sought support from the referees, but in case of their indifference, did not make further attempts to interact with them.

### 8.2. Game Organization

The referees showed the highest quality of interactions with the players in the game organization domain (M = 6.0). The reason for the high assessment of the quality of the referee–player interactions may be the training process, which focuses mainly on the improvement of appropriate signaling, interpretation of players’ behavior and the proper application of the rules of the game. It should be noted here that the quality of interaction of 52% referees in the game organization domain was rated high, and there were no low ratings. This means that the referees were well trained and had skills of whistling (modulation, energy), were able to control situations on the field, discipline and redirect the behavior of players on the field and on the bench, coaching staff, and used the game time effectively.

According to Andersson [22], the pedagogical function of referees is also game organization. Referees are responsible for determining good behaviors, directing the play in relation to the two teams and ensuring the optimal conditions for play. Focusing on the referee–player interactions in the behavior management dimension reveals the referees’ inconsistency. It manifests itself, among others, in situations in which they do not enforce the previously presented expectations, including those related to the behavior of players towards each other. According to Arthur-Banning et al. [41], bad behavior is less likely when punishment is more unavoidable and therefore the referee should be consistent in whistling fouls and other offences. The referees attempted to monitor the behavior of the players, trying to redirect negative behavior, but their strategies and educational methods were not fully effective. There were periods without proactive educational activities of referees, even in situations that required them from the pedagogical point of view. Therefore, during matches, there were episodes of inappropriate sporting behavior. They were usually short and limited to a few players.

Praise or any other form of positive behavioral reinforcement increases the likelihood that the child will repeat the expected behavior. However, the lack of positive sporting attitudes is not only tolerated but sometimes children and young people are even incited by coaches and parents to unsportsmanlike conduct. They want their children to stand out regardless of the means used by them [52]. This can be considered a paradox since adults take measures to prevent children and young people from positive behavior. In situations where adults strengthen and encourage antisocial behavior, the role of the referee is particularly important. Lehman and Riesman [53] point out that the referee is present on the field to not only enforce the rules and regulations of the game on the participants but also to control the coaches and spectators.

The quality of the referee–player interactions in the negative climate dimension was rated high (M = 6.7). This means that few interactions of referees and players were characterized by a negative emotional charge. Other studies have shown that the negative climate of a match can encourage aggressive behavior [15]. Younger athletes often abandon sport because of the negative climate, the disappearance of values such as fair play and sportsmanship, attitudes of competition and winning at all costs, negative experiences in sports relations, strict and inflexible rules that take away the joy of participation [35,36,52,54]. An important reason for children’s abandoning of sports is also the excessive pressure exerted by the coach [15] or parents [20]. If the referee does not build a positive climate, then at least according to the rule primum non nocere, they should not create a negative climate.

### 8.3. Instructional Support

The referees’ job is to explain their decision and correct the misbehavior of players. They also should support players’ learning process by teaching game rules and sporting values. Andersson [22] claim that referees as pedagogue should answer not only to the questions of young players but also explain their decisions to parents and coaches. They can do it during the match or just after. The referees showed a medium quality of interactions with players in the *Instructional Support* domain (M = 5.2). In this domain, the differences in the quality of the interactions of individual referees were also noticeable (SD = 1.8). The referees usually provided clear information about the rules of the game. However, there were situations when they failed to do so, or players did not understand the referee’s instructions, but such cases were very rare. Similar findings were presented by Arthur-Banning, Paisley and Wells [41], who demonstrated that referees in sporting competitions often focused on enforcing the rules, forgetting that they can also be teachers of the game. Young players may not understand the rules of the game and their mistakes. Without an explanation from the referee, they are left with uncertainty and lack of knowledge, which can turn into frustration and aggression. The referee who briefly explains the player’s offence and indicates how he or she may not repeat similar behavior and improve in the future will help him or her better understand the rules of the game, enriching the sporting experience.

## 9. Conclusions

Referees are usually unaware of their pedagogical function and the complexity of the respective responsibilities. They are commonly considered to be ordinary technicians and evaluators of performances in competition [32]. However, the referee is a role model, intentionally or unintentionally influencing the atmosphere of the match and the young players. Especially the youngest children tend to observe and imitate the behavior of adults who are key figures in the sports environment. It follows that if a referee adopts a pedagogical attitude in a conscious and deliberate way, he or she has a chance to significantly influence the intellectual, moral, emotional and social development of the youngest athletes. Based on the results, a postulate can be formulated that referees should consciously perform an educational function in sport for children and young people. In order for this to happen, it is necessary to train them in educational methods and techniques appropriate to the age and needs of the child. The referees will then be prepared to take actions to prevent negative behavior and to encourage prosocial behavior.

The referee, similar to physical education teachers, coaches and parents, should be considered an important link in sports education. The success or failure of a child in sport, understood not only as a sports result but also as the ability to use sports skills and show sports attitudes in everyday life, can be seen as a measure of the skills of the above-mentioned actors. If it is assumed that a referee in children and youth sports represents an educator, teacher and guide for players, the chances of achieving the goals of sports education and shaping a positive image of sports competition will be increased.

## Figures and Tables

**Table 1:** Example of determining the assessment for the dimension of positive climate of the REFASS tool (based on Pianta, Hamre, Mintz 2012).

Positive Climate			
Positive climate reflects the emotional connection and relationships among referees and players, and the warmth, respect, and enjoyment communicated by verbal and non-verbal interactions.			
Indicators	Low (1,2)	Mid (3,4,5)	High (6,7)
Relationships - physical proximity - peer interactions - social conversation	The referee and players appear distant from and disinterested from in one another.	The referee and some players appear generally supportive and interested in one another but the interactions are muted or not representative of the majority of players on the pitch.	There are many indications that the referee and players enjoy warm and supportive relationships with one another.
Positive affect - smiling - laughter - enthusiasm	The referee and players display flat affect and do not appear to enjoy their time on the pitch.	The referee and players demonstrate some indications of genuine positive affect; however, these displays may be brief, muted, or not characteristic of the majority of players on the pitch.	There are frequent genuine displays of positive affect among the referee and players.
Positive communications - positive comments	The referee and players rarely provide positive comments.	The referee and players sometimes provide positive comments; however, these communications may be brief, somewhat perfunctory, or not observed among the majority of players on the pitch.	There are frequent positive communications among the referee and players.
Respect for and recognition of the authority of the referee - respectful language - using first names - warm, calm voice - listening to each other	The referee and players rarely, if ever, demonstrate respect for one another. Competitors do not recognize the authority of the referee, often questioning his or her decision.	The referee and players sometimes demonstrate respect for one another; however, these interactions are not consistently observed across time or players and it happens that the players question the referee’s authority.	The referee and players consistently demonstrate respect for one another. The referee has the authority and his decisions are not called into question.

**Table 2:** The method to determine the assessment of individual dimensions based on their indicators.

Two Indicators *	Three Indicators	Four Indicators	Score	
L, L	L, L, L	L, L, L, L	1	**LOW**
L, M	L, L, M	L, L, L, M	2	
M, L	L, M, M	L, L, M, M L, M, M, M	3	**MID**
M, M	M, M, M L, M, H	M, M, M, M L, M, M, H	4	
M, H	M, M, H	M, M, M, H M, M, H, H	5	
H, M	M, H, H	M, H, H, H	6	**HIGH**
H, H	H, H, H	H, H, H, H	7	

Abbreviations: L = Low; M = Mid; H = High. * in the case of two indicators, the indicator which is higher in the dimension description is more important.

**Table 3:** Descriptive statistics and normality assessment for the ratings of quality of referee–player interactions for individual dimensions observed in soccer referees (n = 25).

Dimension	Mean (SD)	Median	Skewness	Kurtosis	Shapiro-Wilk *p*
Positive Climate	4.0 (1.8)	4.0	0.172	-1.475	0.004
Sensitivity	5.2 (1.5)	5.0	−0.886	1.173	0.016
Behavior Management	4.8 (1.7)	5.0	−0.316	−0.833	0.022
Productivity	6.4 (0.9)	7.0	−1.339	1.038	<0.001
Negative Climate	6.7 (0.9)	7.0	−3.619	13.65	<0.001
Content Understanding	5.2 (1.8)	6.0	−0.632	−0.913	0.002
Quality of Feedback	5.2 (1.9)	6.0	−0.658	−1.148	<0.001

**Table 4:** Results of factor analysis after Varimax rotation (two-factor solution): values represent factor loadings for the assessments.

Variable	Factor 1	Factor 2
Positive Climate		0.789
Sensitivity		0.653
Behavior Management	0.694	
Productivity	0.846	
Negative Climate		0.701
Content Understanding	0.744	
Quality of Feedback	0.715	
Eigenvalue	2.53	2.11
Explained variance	36.2%	30.2%

**Table 5:** Results of factor analysis after Varimax rotation (three-factor solution): values represent factor loadings for the assessments.

Variable	Factor 1	Factor 2	Factor 3
Positive Climate	0.918		
Sensitivity	0.747		
Behavior Management	0.675		
Productivity		0.924	
Negative Climate			0.923
Content Understanding		0.635	
Quality of Feedback		0.646	
Eigenvalue	2.43	1.88	1.19
Explained variance	34.8%	26.8%	17.0%
